# Formative Exploration of the Feasibility of Embedding Community Assets Into Primary Health Care: Barbershop and Place of Worship Readiness in Guyana

**DOI:** 10.1177/21501319221135949

**Published:** 2022-11-14

**Authors:** Sharlene Goberdhan, Reeta Gobin, Olly Perreira, Manoj Sharma, Melissa Ramdeen, Seeromanie Harding

**Affiliations:** 1 University of Guyana, Georgetown, Guyana; 2 King’s College London, London, UK

**Keywords:** community health, community engagement, primary care, readiness assessment, community-primary care partnership, task shifting, community asset-based research, community intervention, community resources, health promotion

## Abstract

**Introduction:**

Community engagement is key to improving the quality of primary health care (PHC), with asset-based interventions shown to have a positive impact on equity and health outcomes. However, there tends to be a disconnect between community-based interventions and PHC, with a lack of evidence on how to develop sustainable community—primary care partnerships. This paper reports on the formative phases of 2 studies exploring the feasibility of embedding community assets, namely places of worship and barbershops, into the PHC pathway for the prevention and control of NCDs in deprived settings. It describes the participatory approach used to map and gather contextual readiness information, including the enablers and constrainers for collaborative partnerships with PHC.

**Methods:**

Grounded in community-based participatory research, we used elements of ground-truthing and participatory mapping to locate and gather contextual information on places of worship and barbershops in urban and rural communities. Local knowledge, gathered from community dialogs, led to the creation of sampling frames of these community assets. Selected places of worship were administered a 66-item readiness questionnaire, which included domains on governance and financing, congregation profile, and existing health programs and collaborations. Participating barbershops were administered a 40-item readiness questionnaire, which covered barbers’ demographic information, previous training in health promotion, and barbers’ willingness to deliver health promotion activities.

**Results:**

Fourteen barbershops were identified, of which 10 participated in the readiness survey, while 240 places of worship were identified, of which 14 were selected and assessed for readiness. Contextual differences were found within and between these assets regarding governance, accessibility, and reach. Key enablers for both include training in health promotion, an overwhelming enthusiasm for participation and recognition of the potential benefits of a community—primary care partnership. Lack of previous collaborations with the formal health system was common to both.

**Conclusion:**

The participatory approach extended reach within underserved communities, while the readiness data informed intervention design and identified opportunities for partnership development. Contextual differences between community assets require comprehensive readiness investigations to develop suitably tailored interventions that promote reach, acceptance, and sustainability.

## Introduction

Communities, comprising the physical and social environment in which groups of people live and work, are critical to improving population health and well-being. Empowering individuals and communities to optimize their own health is a central component of primary health care (PHC),^1^ global commitment to which was reaffirmed through the 2018 Declaration of Astana.^2^ Its other 2 components focus on addressing the broader determinants of health and meeting people’s needs through integrated services. Together, they constitute a whole-of-society approach to healthcare that aims for people-centered, equitable delivery, making PHC a pillar of universal health coverage. Community engagement is recognized as an effective way of improving the quality of PHC as it gives a voice to disadvantaged groups, mobilizes resources and energy, acknowledges the rights of individuals and communities to be actors in the design and delivery of their health care and empowers and enables them to understand their health situation and make informed decisions.^3-5^ Evidence point to its positive impact on an array of health outcomes, including improved access to health services among disadvantage populations, signaling a potential to reduce health disparities.^6,7^

Community-based interventions use various approaches, which can be categorized using Rothman’s typology: community as setting, community as target, community as agent, and community as resource.^8^ The fourth model, “community as resource,” aligns with the belief that community ownership and engagement are pivotal for sustaining community-based health promotion initiatives and aims to mobilize community assets across sectors.^9^ Community assets are available resources that promote health and well-being and protect against negative health outcomes, and include physical places, social networks, practical skills, and interests of local community members.^10,11^ Partnering with community-based organizations and creating opportunities for participation that build on local strengths and assets are actions outlined by the World Health Organization (WHO) to engage communities for the improvement of PHC.^12^ Intersectoral collaboration is therefore a core principle of PHC, with the potential to tackle inequity produced by policy action.^13^

Faith-based organizations have long been recognized as powerful community assets in the promotion of health, particularly in underserved communities, owing to their physical resources, social capital, and their prevalence and accessibility to hard-to-reach groups.^14-16^ Positive health outcomes of interventions in faith-based organizations have been documented in the literature, targeting HIV/AIDS,^17^ chronic diseases,^18^ maternal and child health,^19^ and mental health.^20^ However, women are more likely to be religiously involved and attend church services,^21,22^ and are therefore more likely to benefit from these health interventions than their male counterparts. Barbershops, another community asset, offer the opportunity of meeting men in a place of trust within their community; a place where they frequent, network, and participate in recreation and thoughtful discussions.^23^ Leveraging barbershops for health promotion has already shown promise among marginalized African-American men in the United States, with evidence of a positive impact on blood pressure control^24^ and for the feasibility of training barbers in health promotion.^25^

In low- and middle-income countries (LMICs), where community health workers (CHWs) serve as the link between PHC and the community, task-shifting using non-physician health workers to provide interventions has demonstrated various gains including cost-effectiveness.^26,27^ It is shown to be most successful when there is proper integration with the community and when there is multisectoral engagement.^26^ However, there tends to be a disconnect between community-based interventions and PHC, with fragmented research and limited evidence on social accountability mechanisms for PHC and how to develop sustainable community—primary care interfaces for the achievement of long-term intervention benefits.^26,28^

Guyana is a middle-income country with a population of less than 1 million people.^29^ The poverty rate is 41.2%,^30^ while the unemployment rate in the first quarter of 2020 was 12.8%.^31^ Like many countries within the region, socioeconomic transitioning has resulted in the shifting of disease burden to non-communicable diseases (NCDs). NCDs are responsible for 68% of all deaths, 58% of which occur in people below 70 years; in 2016, Guyana had both the highest NCD mortality rate and the highest premature NCD mortality rate in the Americas.^32^ Progress remains slow with recent predictions indicating that Guyana will not achieve target 3.4 of the Sustainable Development Goals—one-third reduction in premature NCD mortality by 2030.^33^ Men are disproportionately affected, with greater exposure to risk factors and widening disparity between male and female NCD mortality rates.^32^ Tackling the NCD epidemic relies on the tenets of universal health coverage and requires the whole-of-society approach of PHC.^34^

This paper reports on parts of the formative phases of 2 studies exploring the feasibility of embedding community assets, namely places of worship and barbershops, into the PHC pathway for the prevention and control of NCDs in deprived settings. It describes the participatory approach used to map and gather contextual readiness information, including the enablers and constrainers for collaborative partnerships with PHC.

## Methods

The data draws from the 2 studies described. The CONgregations Taking ACTion against NCDs (CONTACT) Study evaluates the feasibility of training congregants in health promotion and screening and explores the development of an interface between places of worship and their nearby PHC center, within 3 Caribbean countries (Guyana, Jamaica, and Dominica).^35^ Guyana was chosen as the primary site for CONTACT due to greater ethic and religious diversity and its economic disadvantage compared to the other sites. The Barbershop Study, an extension of CONTACT in Guyana, assesses the preparedness of barbershops for the promotion of men’s health, an intervention that would see barbers being trained in NCD-related health promotion and screening with oversight from the nearby PHC center.

### Setting

Guyana is divided into 10 administrative regions, with Regions 1 to 6 and 10 located on the flat coastland. Region 3, Essequibo Islands-West Demerara, and Region 5, Mahaica-Berbice, were selected for participation in the CONTACT Study based on ease of access, relative deprivation, population size, and adequate representation of the nation’s 3 major religions (Christianity, Hinduism, and Islam).^35^ These regions are rural with few semi-urban areas and are major agricultural producers. Each region is divided into small villages set along a main road. Groups of 3 to 4 contiguous villages form officially defined neighborhoods, which are managed by Neighborhood Democratic Councils. Region 4, Demerara-Mahaica, which houses the capital city, Georgetown, was selected as the site for the Barbershop Study as it is home to 41% of Guyanese men^36^ and is representative of Guyana’s largest ethnic groups—Afro-Guyanese, Indo-Guyanese, and Mixed-Guyanese. Apart from Georgetown, which is a gridwork of communities, the remainder of the region has a geographic layout similar to Regions 3 and 5.

Nineteen health centers were identified as possible sites for the CONTACT intervention, each of which serves a demarcated catchment area. Each area was surveyed to identify all places of worship. Five health centers were identified by the Men’s Health Department of the Ministry of Health as offering or intending to offer men’s health services. Nineteen villages from these catchment areas (8 urban, 1 suburban, and 10 rural) were sampled and surveyed to identify all barbershops.

### Identifying community assets

Due to a lack of business lists and registries, we used ground-truthing, a method of physically verifying attributes of a community,^37^ to create sampling frames of places of worship and barbershops. Printed maps of the regions were obtained from the Guyana Lands and Surveys Commission, which, together with Google Maps, guided us through the communities. Exploration was done by car and on-foot due to poor road conditions in many rural areas. Traveling between communities required various modalities, including a ferry and a horse-drawn cart. Google Maps provided less details for the rural regions, resulting in the combination of ground-truthing with elements of participatory mapping. Participatory mapping is an interactive approach that draws on local knowledge, enabling participants to create visual and non-visual data to explore social problems, opportunities, and questions.^38^

Between August 2016 and June 2017, we met with staff of the Neighborhood Democratic Councils located within identified catchment areas to generate lists of places of worship based on their local knowledge. Using small base-maps of the surrounding areas, the staff collectively mapped the locations of the listed places of worship, providing additional information such as recommended routes and relevant contact details, if known. If these base-maps were unavailable, rough sketches were made on plain sheets of paper. We then used these augmented maps to explore the surrounding communities to identify and gather contextual information on all places of worship. Similarly, in May 2019, we canvassed the 19 randomly selected villages guided by Google Maps and the printed map, to identify and obtain contextual information on all barbershops.

Places of worship were recognized by signboards or the local architectural profile of religious buildings; churches were identified by their crosses, steeples, and ventilation blocks, mosques by their white and green domed roofs, and mandirs by their idols and colorful flags. Barbershops were identified by a signboard or barber’s pole, and in more rural areas, they were recognized as small, enclosed spaces in villagers’ yards.

### Contextual information

**Figure 1.**
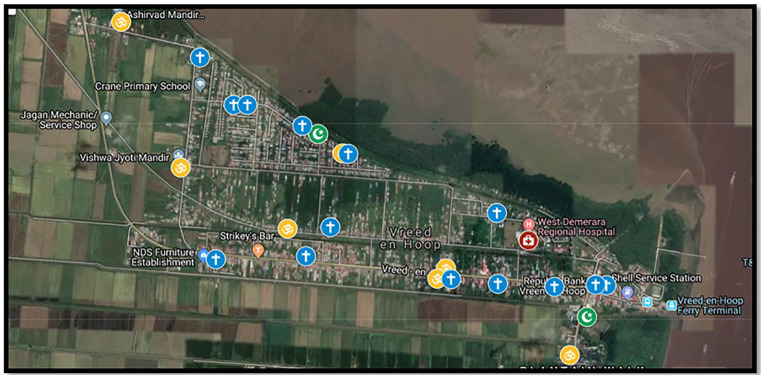
Example of a satellite map augmented by participatory mapping, illustrating the Vreed-en-Hoop PHC center and identified places of worship. The red point represents the PHC center, blue crosses represent churches, yellow oms represent mandirs, and green crescents represent mosques.

Once a place of worship was identified, we sought to obtain contextual information, including but not limited to congregation size, timing of services, and willingness to participate in the CONTACT intervention. Community engagement, which included walk-along dialogs with passers-by and roadside vendors, was critical in identifying key informants and obtaining information, and in locating the informal “bottom-house churches” that lacked permanent, physical structures. Coordinates of all places of worship were identified on Google Maps and a digital map was created to display their geographical layout relative to the health centers (see Figure 1). We used the information obtained to generate a sampling frame from which the intervention participants were selected. A 66-item place of worship readiness questionnaire, adapted from the instrument created for the Civil Society NCD Regional Status Report,^39^ was administered to the leaders of the selected places of worship. Questionnaires included domains on governance and financing, congregation profile, and existing health programs and collaborations. Once barbershops were identified, a 40-item readiness questionnaire (Supplemental Material, S1) was administered to the owner or manager. Questions covered barbers’ demographic information, details of any prior training in health promotion and barbers’ willingness to deliver health promotion activities. In both studies, informed written consent was obtained and records were made of location and accessibility, space and facilities on the premises, and interactions with community members.

## Results

The combination of participatory mapping and ground-truthing resulted in the coverage of 3300 km and identification of 240 places of worship across the 19 catchment areas: 169 churches, 52 mandirs, and 39 mosques. Fourteen places of worship in Region 3 (5 mandirs, 5 mosques, and 4 churches) were later selected for the intervention and administered a readiness questionnaire. Fourteen barbershops were identified in the 19 sampled villages, of which 10 (5 urban, 1 suburban, and 4 rural) consented to participate in the readiness survey. Table 1 presents a summary of key domains included in the barbershop and place of worship readiness assessments.

**Table 1.** Readiness Assessment of Barbershops and Places of Worship to Be Embedded in the Primary Care System.

Characteristic	Barbershops (n = 10)	Places of worship (n = 14: 5 mandirs, 5 mosques, 4 churches)
Geography	5 urban, 1 suburban, 4 rural	All rural
Governance	All barbershops independently owned, with decisions made by the owner; 1 has a manager.	13 have administrative bodies responsible for decision making; religious leader responsible for decision making in 1 and 4 report to a central organization at least annually.
Infrastructure, accessibility	Limited space inside shops; can accommodate small health corner but cannot provide privacy. Open spaces beside shops may facilitate temporary structures (eg, tents). 8 located along or nearby to main roads/bus routes and 2 within walking distance.	Varying sizes, but all able to provide some space for health-related activities. 7 located along bus route, 5 located walking distance from bus route and 2 require taxi/private transportation.
Member/client profile	All shops cater to men of all ages. Number of regular clients range from 20 to >200; 9 also offer services to women. 7 have clientele with evenly distributed ethnicities, 2 service predominantly Indo-Guyanese, and 1 services predominantly Afro-Guyanese.	Number of regular members range from 20 or fewer in 2 places of worship to >60 persons in 7 places of worship. 8 have a greater proportion of female congregants while 4 have more male congregants. 4 have a majority of young congregants, 1 has an older congregation, 2 have majority middle-aged congregants, and the others are mixed.
Reach	7 shops have majority of their clients from the surrounding neighborhood, 1 has majority of clients from villages up to 77 km away and 2 have clients from different areas along the coast.	Most of the members for 13 places of worship reside in the surrounding communities. Majority of the congregation for 1 place of worship reside in other villages along the coast.
Training in health	3 interviewees with training in health promotion, mostly related to hygiene	9 have congregants trained in health (total of 9 nurses and 9 doctors)
History of collaborations with health organizations	No prior collaborations	2 with past collaborations with Ministry of Health to host one-off outreaches/HIV testing and counseling
Perspectives on participation/health promotion	8 willing to provide health information to clients, of which 7 are willing to participate in a 2-week training. 8 believe clients would be interested in receiving health information.	All willing to participate and believe congregation would be supportive of a health advocate trained from among them

### Barbershop and place of worship readiness: Potential enablers and constrainers

Readiness assessments included places of worship from all 3 major religions and barbershops with varying capacities and price points. The barbershops had operating hours most days, with longer hours on the weekends to reach more people, whereas the places of worship mostly operated for scheduled services, prayers, and functions. Congregations were comprised of more women, except for 4 mosques that had a greater proportion of men. Most places were well-established, with only 1 barbershop being in operation for less than 2 years. Governance structure of the barbershops simplified decision-making as approval from a board or parent organization would be unnecessary, unlike the places of worship. Places of worship had more space to accommodate health-related activities including cooking facilities; however, barbershops were equipped with entertainment devices which may allow digital screens and health corners (8 shops had at least 1 television and 4 had Wi-Fi). Both groups thought training in health promotion and provision of equipment for basic health measures would be enablers; barbers also believed endorsement from local celebrities would encourage utilization of the intervention.

Generally, interviewees appreciated the potential benefits of health promotional activities to their communities. Some barbers also recognized the potential benefits to themselves and displayed enthusiasm for participation, with one saying, *“I can’t read and write good, but I’m interested in learning, and if y’all start, must remember to call me.”* Notably, most interviewees were willing to participate without any financial compensation. One barber suggested a payment of $30 000 GYD ($150 USD) per month; others thought it was unnecessary and that any financial compensation would be a “donation” or a “blessing.” Two religious leaders thought funding would be needed for the health advocates and for the place of worship. One barber was concerned that spending time on health promotion would interfere with his business; nevertheless, most agreed that playing health videos and talking to clients during appointments would be the best methods of delivering health information. Religious leaders thought community support, good leadership, and mutual trust and respect would be important for a successful community—primary care partnership. Most of these community assets had no history of collaborations with the formal health care system, and for those that did, these activities were usually one-off. A few also had regular members residing outside the catchment area of the local health center, a potential constrainer to these persons accessing health services if referred through the intervention.

## Discussion

Grounded in community-based participatory research, we collaborated with urban and rural communities to locate and gather contextual information on places of worship and barbershops, obtain community insights and identify potential enablers and constrainers to the embedding of these community assets in PHC. Readiness assessments revealed contextual differences between the two: places of worship usually have an administrative body, adequate space for health-related activities and a greater proportion of female congregants, but are only open for scheduled services and functions; barbershops are independently owned, cater mostly to men and are open most days of the week, but have limited space for health promotion activities. Key enablers for both include training in health promotion, an overwhelming enthusiasm for participation and recognition of the potential benefits of a community—primary care partnership. However, with little to no history of collaborations with the formal health system, they lack a foundation for building this partnership.

Both studies were strengthened using a participatory approach, which served to build trust and relationships with the communities and leverage villagers’ local knowledge. Their willingness to collectively share information resulted in the inclusion of small, informal places of worship and barbershops that may be absent from official lists. The on-the-ground participatory approach was time- and resource-intensive amid the contextual difficulties of locating these assets in remote communities. However, it extended the reach of subsequent interventions in communities with limited access to health care and helped to obtain representative perspectives. Due to contextual differences, even between places of worship of the same religion, caution should be taken when making generalizations about levels of readiness.

Little evidence exists for assessing the readiness of places of worship and barbershops for health promotion interventions, with the available literature focused on African-American Christian churches in the United States. Brand and Alston^40^ explored predictors of readiness to engage African-American churches in health and found that physical structure and partnerships with health organizations were considered to be important, with varied opinions on the necessity of funding, similar to our findings. Having personnel to coordinate health activities was also seen as being essential for success.^40^ Pichon et al^41^, who reported on the factors influencing church readiness for HIV prevention and treatment activities, recognized the importance of this human resource, in addition to the blessings and authorization of the pastor. While examining the integration of community-based health promotion programs and PHC, Leppin et al^28^ found that community stakeholders possessed great enthusiasm and recognized the value of these programs, while also believing that community resources were vital for effective PHC. This aligned with the willingness displayed by our participants, suggesting an ease of acceptance by the direct beneficiaries of such community asset-based interventions. Additionally, authors of a qualitative systematic review of barber-administered health programs reported on issues of time constraints and competing priorities, concerns also expressed by one of our barbers, and highlighted the importance of commitment recognition and incentives, monetary or otherwise, in a sustainable, mutually-beneficial partnership.^22^

Community engagement is the first step toward building sustainable partnerships between the community and PHC. A participatory, asset-based approach facilitates meeting people in places of trust, thereby encouraging participation and social accountability, and provides the contextual information needed to understand community needs and motivations, inform policy and practice and tailor interventions. These are all key factors in the enhancement of PHC, which is of great urgency amid the healthcare crisis created by the COVID-19 pandemic. Now critical than ever, opportunities should be explored for the shifting toward health governance models that recognize the value of locally produced knowledge whilst accounting for the complexities and importance of decentralizing healthcare from central government to local entities.

## Conclusion

Using a participatory approach to leverage local knowledge is essential when conducting community-based research in a resource-poor setting. Community enthusiasm and recognition of direct benefits are potentially key enablers for a community—primary care partnership. Contextual differences between community assets require comprehensive readiness investigations to develop suitably tailored interventions that promote reach, acceptance, and sustainability.

## Supplemental Material

sj-pdf-1-jpc-10.1177_21501319221135949 – Supplemental material for Formative Exploration of the Feasibility of Embedding Community Assets Into Primary Health Care: Barbershop and Place of Worship Readiness in Guyana Click here for additional data file. Supplemental material, sj-pdf-1-jpc-10.1177_21501319221135949 for Formative Exploration of the Feasibility of Embedding Community Assets Into Primary Health Care: Barbershop and Place of Worship Readiness in Guyana by Sharlene Goberdhan, Reeta Gobin, Olly Perreira, Manoj Sharma, Melissa Ramdeen and Seeromanie Harding in Journal of Primary Care & Community Health
