# Supplementary material for: Formative Exploration of the Feasibility of Embedding Community Assets Into Primary Health Care: Barbershop and Place of Worship Readiness in Guyana
Source: J Prim Care Community Health. 2022 Nov 14;13:21501319221135949. doi: 10.1177/21501319221135949 (PMC9666845; doi:10.1177/21501319221135949)
Supplement: sj-pdf-1-jpc-10.1177_21501319221135949 – Supplemental material for Formative Exploration of the Feasibility of Embedding Community Assets Into Primary Health Care: Barbershop and Place of Worship Readiness in Guyana [file sj-pdf-1-jpc-10.1177_21501319221135949.pdf]

(CONTACT Team) - Barbershop Survey

Interviewer initials ____________________ Date ______________________

1. Name of Barbershop ____
2. Name and designation of interviewee ____
3. Address & Contact number(s) ____

____

4. Urban [ ] Rural [ ]
5. How long has your barbershop operated at this location? ________________________________
6. Is this barbershop registered with any official/government agency? Yes [ ] No [ ]
7. If yes to (6), which? ____
8. Are you aware of the existence of the Guyana Barbers' Association? Yes [ ] No [ ]
9. If yes to (8), are you or this barbershop a member? Yes [ ] No [ ]
10. How many days a week does the shop open? ____________________ (*write number*)
11. What are your opening hours on weekdays? ___________to___________ (*24-hr clock)*

    On weekends? ___________to___________ Other? ___________ to ___________
12. What are your busiest times during
    a. The day _____________________ b. The week _____________________
13. How many barbers work in this shop? __________________
14. Please state how many are
    a. Full-time ______________
    b. Part-time ______________
15. Can you state how many of them
    a. Completed secondary education _______________
    b. Completed tertiary education _____________
    c. Completed technical schooling _____________

16. Please state the highest level of education you completed ______________________________

17. Can you please list the services that you usually offer? (*may need prompting to understand question*)

____

____

18. Please state the average cost of a haircut. *(Request and record price list if available.)*

____

19. Think of the past week. On average, what was the total number of clients who accessed services

provided by you _______________? Your shop _____________?

20. On average, how often do regular clients visit your shop? (*May need prompting, explain 'regular'*)

[ ] >once per week [ ] once per week [ ] twice per month [ ] once per month

[ ] other __________________________ (*example: every 3 weeks, every 5 weeks, etc*)

21. Are most of your clients from the surrounding neighbourhoods? Yes [ ] No [ ]

22. If no to (21), please state where they come from

____

23. Are <u>all</u> of your clients men? Yes [ ] No [ ]

24. If no to (23), approximately what proportion are women? _____________ (*may need prompting)*

25. What is the age range of the majority of your customers?

[ ] 18 – 24 [ ] 25 – 34 [ ] 35 – 44 [ ] 45 – 54 [ ] 55 – 64 [ ] 64+ [ ] All ages

26. Can you say which ethnic group most of your customers come from? (*may need prompting)*

[ ] Afro-Guyanese [ ] Indo-Guyanese [ ] Mixed [ ] Other

[ ] Evenly distributed [ ] Prefer not to state [ ] Don't know

27. On average, how much time would you say each client has to wait before it is his/her turn?

____ (*minutes*)

28. How do clients pass the time while waiting?

____

____

29. Does your shop have a functional

    [ ] Radio [ ] Television set [ ] DVD player [ ] Wi-Fi

30. Have you or your barbers had any special training in health or health promotion? [ ] Yes [ ] No
31. If yes, please state what kind(s) of training.

    ____

    ____

32. Do barbers and clients normally talk about health-related topics in the shop? Yes [ ] No [ ]
33. If yes, what kind of topics?

    ____

    ____

34. Do you think your customers would be interested in receiving health information and basic health testing? Yes [ ] No [ ] Not sure [ ]
35. Do you think the barbers in this shop would be interested in providing health promotion to their clients? Yes [ ] No [ ]
36. If yes,
    a. Would you be able to participate in a two-week training on health promotional activities? *(Explain that the training would take place at timings most convenient to barbers*.)

       Yes [ ] No [ ]

    b. What type of health promotion activities do you think would be possible at your barbershop?

       (i) **Education** about (*tick*)

       ☐ Diabetes ('Sugar')
       ☐ High blood pressure
       ☐ Heart disease
       ☐ Cancer
       ☐ Smoking
       ☐ Excessive alcohol use
       ☐ Mental health (*give examples*)
       ☐ Men's health (*give examples*)
       ☐ Diet and exercise

       (ii) **Screening** for (*tick*)

       ☐ High blood pressure
       ☐ High blood sugar
       ☐ Overweight
       ☐ Mental ill health e.g. depression

       (iii) A health corner with information on health and health services available to men?

       Yes [ ] No [ ]

(iv) monthly clinics run by a nurse or community health worker from the nearest clinic? *(Explain that the space for the clinic can be inside or outside. Set up will be done by health centre and other staff. May need to explain what clinic would entail.)* Yes [ ] No [ ]

c. Would your barbershop be willing to advertise these clinics on a billboard or digital signboard, if provided? Yes [ ] No [ ]

37. What methods of sharing health information with clients do you think would be most ideal in your barbershop? *(You can select more than one)*

    [ ] Distribute pamphlets [ ] Posters displayed in your shop [ ] Talk with clients during their appointments [ ] Digital displays of services [ ] Show a video about health

38. What would assist barbers to support health promoting behaviours among men?

    [ ] Monthly meetings with health centre staff
    [ ] Easy reading material on health
    [ ] Local sports figure/celebrity advertising the service
    [ ] Training of barbers
    [ ] Financial compensation. If so, how much (per month) ___________________________________
    [ ] Support of community organisations. If so, which and how can they be used?

    ____

    ____

    ____

39. What would make it difficult for barbers to encourage health promoting behaviours among men?

    [ ] Reduced time spent on barbershop business
    [ ] Interference with customer relationships
    [ ] Reduced chatting time about usual activities
    [ ] Push customers to other barbershops
    [ ] Lack of private space in shop for clinics
    [ ] Lack of space for billboards
    [ ] Lack of space outside for monthly clinics
    [ ] Poor relationship between customer and health centre staff

40. Have you or your barbershop ever participated in any activities with any of the following organisations? [ ] Redthread [ ] UNICEF [ ] PAHO [ ] Local charities/organisations

    [ ] Other, please state ____

    If yes to any, please state when, and what the activity was about.

    ____

    ____
